# Tumor-microenvironment responsive nano-carrier system for therapy of prostate cancer

**DOI:** 10.1007/s10856-023-06749-9

**Published:** 2023-09-21

**Authors:** Lujing Li, Renjie Li, Jiachun Li, Jiyi Yao, Qingyuan Zhang, Qiao Ji, Zuofeng Xu

**Affiliations:** 1 Department of Ultrasound, The Seventh Affiliated Hospital, Sun Yat-sen University, Shenzhen, 518107 China; 2 Department of orthopedics, The Seventh Affiliated Hospital, Sun Yat-sen University, Shenzhen, 518107 China; 3 Department of Ultrasound, Sun Yat-sen Memorial Hospital, Sun Yat-sen University, Guangzhou, 510120 China; 4 Department of Gastrointestinal surgery, The Seventh Affiliated Hospital, Sun Yat-sen University, Shenzhen, 518107 China

## Abstract

Poor selectivity, low bioavailability and serious systemic side-effects have limited the application of traditional chemotherapy method for treatment of prostate cancer. Stimuli-responsive drug delivery systems for chemotherapy are mainly based on the unique characteristics of tumor microenvironment. In this study, the GSH-sensitive poly-TTG-SS@DTX NPs (DTX-loaded poly-Tetraethylene glycol nanoparticles) were designed and synthesized, which were characterized with nanosized diameter (92.8 ± 2.5 nm) and negatively charged surface charge (−24.7 ± 5.56 mV). Experiments in vitro showed that poly-TTG-SS@DTX NPs had good compatibility to healthy cells and strong anti-tumor effect because of rapid and sustained drug release of DTX from poly-TTG-SS@DTX NPs under the tumor-microenvironment condition. The cellular activity remained greater than 90% when the concentration of poly-TTG-SS NPs reached as high as 100 µg/mL treated on healthy cells. The killing effect of DTX loading NPs group on C4-2 cells was stronger than that of free anti-tumor drug and free DTX combined with the blank nano-carrier (25.21% vs 19.93% vs 20.96%). In conclusion, poly-TTG-SS@DTX NPs may provide a new therapeutic strategy for the chemotherapy of prostate cancer.

**Graphical Abstract:**

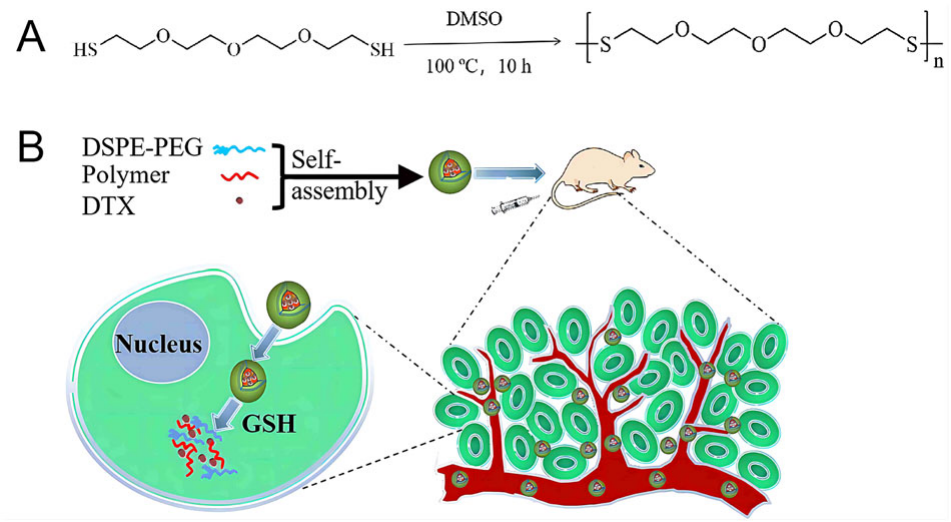

## Introduction

Cancer has been a major public health problem worldwide and severely threatens human life for decades [1, 2]. Prostate cancer is the most common malignancy and causes the second greatest number of deaths diagnosed in men [1]. Conventional chemotherapy is one of the most common treatments for castration-resistant prostate cancer. Docetaxel (DTX) is the first-line chemotherapy drug in the guideline [3]. However, poor selectivity, low bioavailability and serious systemic side-effects have limited the application of traditional chemotherapy method [4].

Over the past few decades, nanoparticle (NP) -mediated drug delivery system has been applied for efficient chemotherapeutic drugs delivery and treatment of cancer, some of which have been approved for clinical translation [5–7]. However, the traditional nano-carriers still face a lot of challenges, such as low drug loading, potential toxicities and non-active drug release, which result in unsatisfactory treatment effect. In this way, stimuli-responsive drug delivery systems have been developed [8–10]. Stimuli-responsive drug delivery systems for chemotherapy are mainly based on the unique characteristics of tumor-microenvironment, such as the acidic pH value, high concentrations of ROS, esterase and glutathione (GSH) [11]. The intracellular concentration of GSH (up to 2–10 mM) is significantly higher than normal blood and its extracellular concentration (2–20 µM) [12]. Studies have also shown that the intracellular concentration of GSH is significantly higher in the prostate cancer cells, and the reduction state is further improved with the progression of prostate cancer [13, 14]. Therefore, the marked difference in reduction potential between prostate cancer and normal tissues has been exploited as a promising reduction-responsive drug delivery strategy for cancer therapy [15, 16]. Disulfide linkages have been utilized to construct the reduction-responsive nano-carriers. The interaction between disulfide bonds and the high concentration of intracellular GSH enables the rapid release of drugs from nano-carriers, and the disulfide bonds remain stable under normal physiological conditions [17]. In our study, polymer with GSH-sensitivity was designed and synthesized. Anticancer drug DTX was loaded in the self-assembled polymer.

## Materials and methods

### Materials

Tetraethylene glycol (TTG), trimethylamine (TEA), tetrahydrofuran (THF) (3-(4,5-Dimethylthiazol-2-yl)-2,5-diphenyltetrazolium bromide (MTT), 1,2-distearoyl-sn-glycero-3-phosphoethanolamine-N-[methoxy(polyethylene glycol)-3400] (DSPE-PEG3400), Dil, Coumarin-6 (C6) were provided by Sigma Aldrich Co. Ltd. The Annexin V-FITC/PI apoptosis detection kit was obtained from Invitrogen. Dulbecco’s modified Eagle’s medium (DMEM), Roswell Park Memorial Institute (RPMI) 1640 medium, trypsin-EDTA, fetal bovine serum (FBS), penicillin–streptomycin solution, phosphate-buffered saline (PBS), and water were purchased from GibcoR.

### Synthesis of poly-TTG-SS

The polymer poly-TTG-SS was obtained by the following approach. Briefly, 600 μL of TTG (10 mmol, 2260 mg) was dissolved in 1.2 mL of TEA (16.89 mmol, 1700 mg) under stirring. Afterwards, 3.5 mL of 3 wt% hydrogen peroxide (H_2_O_2_) was added dropwise for over 5 min. The polymerization could also be carried out in water. After 10 min of reaction, the molecular weight of the homopolymer was determined by GPC (Agilent, PL gel columns 5 µM MIXED-C).

### Synthesis of poly-TTG-SS NPs and drug-loaded poly-TTG-SS NPs (poly-TTG-SS@DTX NPs)

The method of nano-precipitation was conducted to prepare poly-TTG-SS NPs and poly-TTG-SS@DTX NPs. DSPE-PEG3400 (0.4 mg, 4 mg/mL) and poly-TTG-SS (2 mg, 20 mg/mL) were dissolved in DMSO (100 μL), respectively. Then the mixture was dripped to the water phase (9.0 mL), followed by 1 mL of 10× PBS. The poly-TTG-SS NPs could be self-assembled in water. Organic solvent was removed through the ultrafiltration in deionized water at 25 °C for 3 times. The prepared NPs was stored at 4 °C.

To synthesize poly-TTG-SS@DTX NPs, DSPE-PEG3400 (0.4 mg, 4 mg/mL), poly-TTG-SS (2 mg, 20 mg/mL) and DTX (0.5 mg, 5 mg/mL) were separately dissolved in DMSO (100 μL) and mixed well. After the centrifugation for 3 times, poly-TTG-SS@DTX NPs were obtained for further experiments.

### Characterization of poly-TTG-SS and poly-TTG-SS NPs

The ^1^H NMR (nuclear magnetic resonance) spectra of the polymer was characterized using Ascend TM 500 spectrometer (Bruker, Germany). The sizes, PDI (polydispersity index), and *ζ*-potential values of blank and DTX-loaded NPs was recorded by DLS (dynamic light scattering) on the Zetasizer (Nano ZS, Malvern Co., UK). TEM (transmission electron microscopy) was applied to observe the morphology on the JEM-1400 Plus (JEOL, Japan). The long-term stability of poly-TTG-SS NPs was determined in different dispersion media by DLS. Typically, 1 mg/mL of poly-TTG-SS NPs were dispersed in PBS (pH 7.4), FBS and RPMI1640 containing 10% FBS and the particle diameters were monitored at preset time points.

HPLC (high-performance liquid chromatography) was utilized to calculate the drug loading content (DLC) and encapsulation efficiency (EE) of poly-TTG-SS@DTX NPs on Shimadzu LC-20AD (Kyoto, Japan). The calculation formula was as follows:

1 $$DLC( \% )=\frac{{\rm{The}}\,{\rm{weight}}\,{\rm{of}}\,{\rm{DTX}}\,{\rm{in}}\,{\rm{NPs}}}{{\rm{The}}\,{\rm{weight}}\,{\rm{of}}\,{\rm{the}}\,{\rm{whole}}\,{\rm{NPs}}}\times 100 \%$$

2 $$EE( \% )=\frac{{\rm{The}}\,{\rm{weight}}\,{\rm{of}}\,{\rm{DTX}}\,{\rm{in}}\,{\rm{NPs}}}{{\rm{The}}\,{\rm{total}}\,{\rm{input}}\,{\rm{weight}}\,{\rm{of}}\,{\rm{DTX}}}\times 100 \%$$

### Cell culture

C4-2 cell line (human prostate cancer), 3T3 cell line (mouse embryonic fibroblast cell line) and RWPE-1 (Cat. #CRL-11609, the human normal prostate epithelial cell line) were obtained from ATCC (American Tissue Culture Collection) and separately cultured in RPMI 1640 medium and DMEM medium containing 10% FBS at carbon dioxide incubator with 5% CO_2_ at 37 °C.

### In vitro GSH-responsive release of DTX from DTX-loaded poly-TTG-SS NPs

The GSH-responsive release of DTX from poly-TTG-SS@DTX NPs was evaluated by the dialysis method. To be specific, 1 mL of poly-TTG-SS@DTX NPs solution (equivalent to 2 mg of DTX) was loaded into dialysis tubes (MWCO = 3500). Subsequently, dialysis tubes were placed in 20 mL of PBS containing 0.1% Tween 20 (m/v) using different concentrations of GSH (0, 1 μM, 10 μM, 1 mM, 10 mM) as the dialysis fluid. The dialysis bags were put in a shaking incubator under a rotation rate of 100 rpm for about 72 h. At pre-determined time points, the released amount of DTX was sampled and determined by HPLC. The compositions of the mobile phase were H_2_O/CH_3_CN (40/60, v/v).

### In vitro Cellular Uptake of poly-TTG-SS NPs

To quantitatively analyze the cell uptake of poly-TTG-SS NPs for antitumor drugs, flow cytometry (FCM, Beckman Coulter, USA) was conducted and fluorescent probe molecule coumarin 6 (C6) was used instead of DTX. C6-loaded NPs (poly-TTG-SS@C6 NPs) were prepared by nano-precipitation method (equivalent concentration of C6: 1 µg/mL). C4-2 cells were seeded in 6-well plates at the density of 2 × 10^5^ cells per well and cultivated overnight. Then the medium was further replaced by complete 1640 medium containing different formulations (C6 or poly-TTG-SS@C6 NPs) and incubated for 1 h, 3 h or 6 h, respectively. Finally, the fluorescence intensity was analyzed by FCM at the FITC channel.

The direct cellular uptake of poly-TTG-SS NPs in C4-2 cells was observed by CLSM (confocal laser scanning microscopy) on ZEISS880 (Germany). Fluorescent probe Dil was used instead of DTX. The reason was that DTX do not give off light and cannot be observed. Dil-loaded NPs (poly-TTG-SS@Dil NPs) were prepared by nano-precipitation method. In detail, DSPE-PEG3400 (0.4 mg, 4 mg/mL), poly-TTG-SS (2 mg, 20 mg/mL) and Dil (0.5 mg, 5 mg/mL) were separately dissolved in DMSO (100 μL) and mixed. After the centrifugation for 3 times, poly-TTG-SS@Dil NPs were obtained for further experiments. C4-2 cells (density of 2 × 10^5^/well) were seeded on confocal dishes and cultured overnight in the cell incubator. Then the cells were incubated with medium containing Dil (concentration: 1 µg/mL) or Dil-loaded NPs (equivalent concentration: 1 µg/mL) for another 1 h, 3 h, 6 h. Next, the prepared 4% paraformaldehyde solution was used to fix the cells for 15 min. Thereafter, the cytoskeleton was stained by FITC-phalloidin for 30 min and nuclei was stained by DAPI for 10 min. Subsequently, the fluorescence pictures were acquired by selecting the corresponding channel to observe C6, cytoskeleton and nucleus. Additionally, the uptake of poly-TTG-SS NPs by RWPE-1 cells was also detected by FCM and CLSM.

### Cytotoxicity assays

The anticancer activity of poly-TTG-SS@DTX NPs and cytotoxicity of poly-TTG-SS NPs against human prostate cancer cells (C4-2 cells), mouse embryonic fibroblast 3T3 cells and human normal prostate epithelial cells (RWPE-1) were evaluated by MTT. Specially, 3T3 cells, RWPE-1 cells and C4-2 cells were inoculated in 96-well plates (6000/well) for 12 h. Then the medium was removed and replaced by complete medium containing different formulations: blank NPs (0.05, 0.25, 0.5, 1.25, 2.5, 5, 10, 25, 50, 100 µg/mL), free DTX, DTX+poly-TTG-SS NPs or drug-loaded poly-TTG-SS@DTX NPs (equivalent concentrations of DTX: 0.001, 0.01, 0.05, 0.1, 0.25, 0.5, 1, and 10 µg/mL). After incubation for 48 h, 20 µL of MTT solution (5 mg/mL) was added to each well for 4 h cultivation. Next, the formed formazan was dissolved after adding 150 µL of DMSO. Finally, the absorbance (OD) value of each well was detected by using the microplate (Varioskan Flash, Thermo, Waltham, MA; wavelength: 490 nm).

### Flow cytometry analysis of cell apoptosis

To further evaluate the antitumor effect of DTX-loaded NPs against C4-2 cells in vitro, flow cytometry was used for analysis of cell apoptosis. C4-2 cells were seeded in the 6-well plates at the density of 2 × 10^5^ cells per well and cultured overnight in the CO_2_ cell incubator, followed by co-incubating with different groups for 48 h. The groups were divided into control group (normal medium), poly-TTG-SS NPs group, free DTX group, DTX+ poly-TTG-SS NPs group and drug-loaded poly-TTG-SS@DTX NPs group. Then cells were resuspended in 500 µL of binding buffer mixture in a flow tube (containing 5 µL Annexin V-FITC and 5 µL PI). The reaction was performed in the dark for 30 min, and then detected by flow cytometer.

## Results

### Preparation and characterization of poly-TTG-SS, poly-TTG-SS NPs and poly-TTG-SS@DTX NPs

**Fig. 1:**
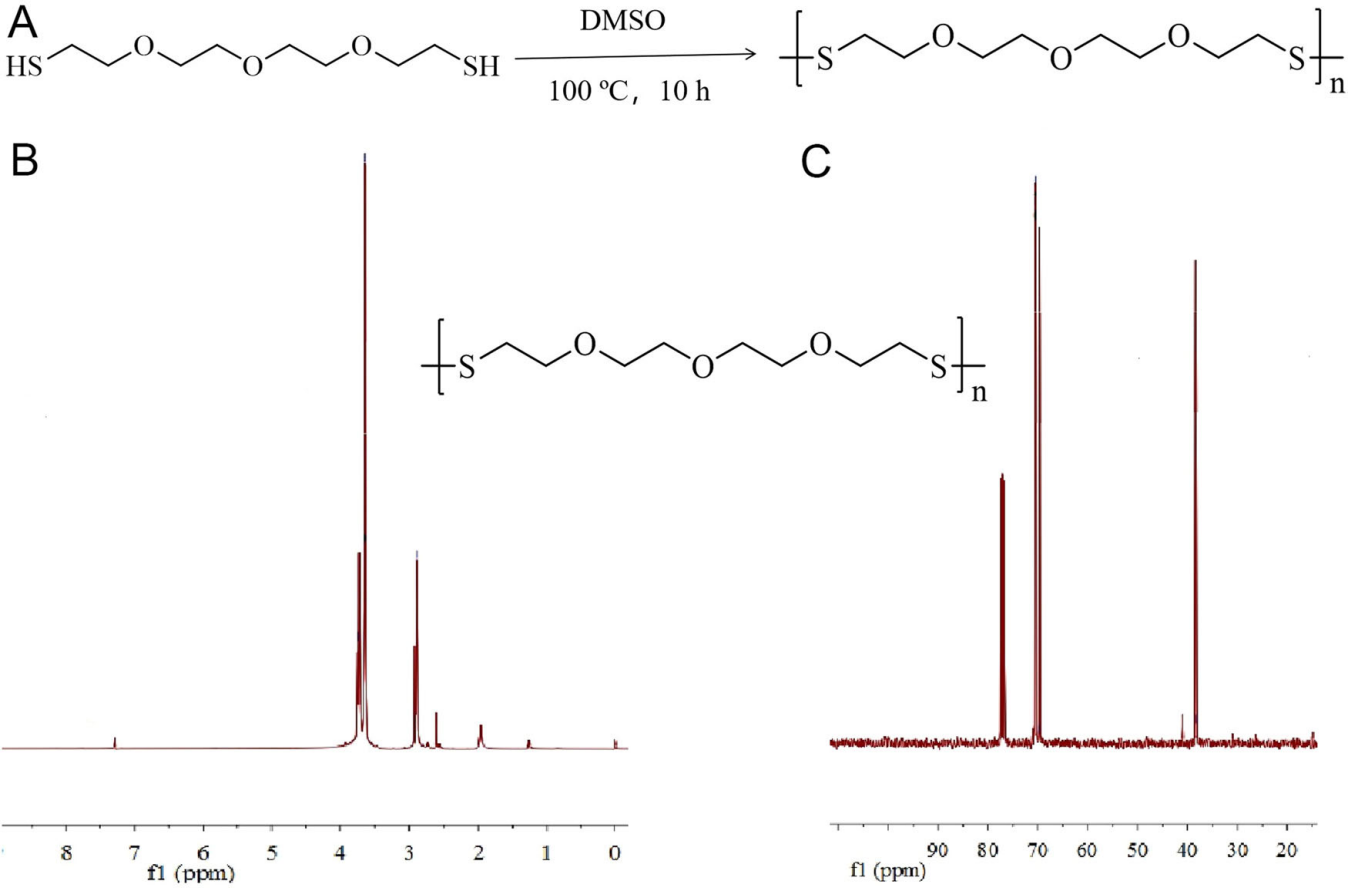
The synthesis and characterization of the poly-TTG-SS. **A** Synthetic process of the poly-TTG-SS. **B** ^1^H-NMR spectrum of poly-TTG-SS. **C** ^13^C-NMR spectrum of poly-TTG-SS

Poly-TTG-SS was prepared and the structure was characterized by ^1^H NMR and ^13^C-NMR as shown in Fig. 1. The disappearance of skeletal vibration of sulfydryl demonstrated that the disulfide bond was successfully synthesized. The molecular weight of the homopolymer was 1365 Da.

**Fig. 2:**
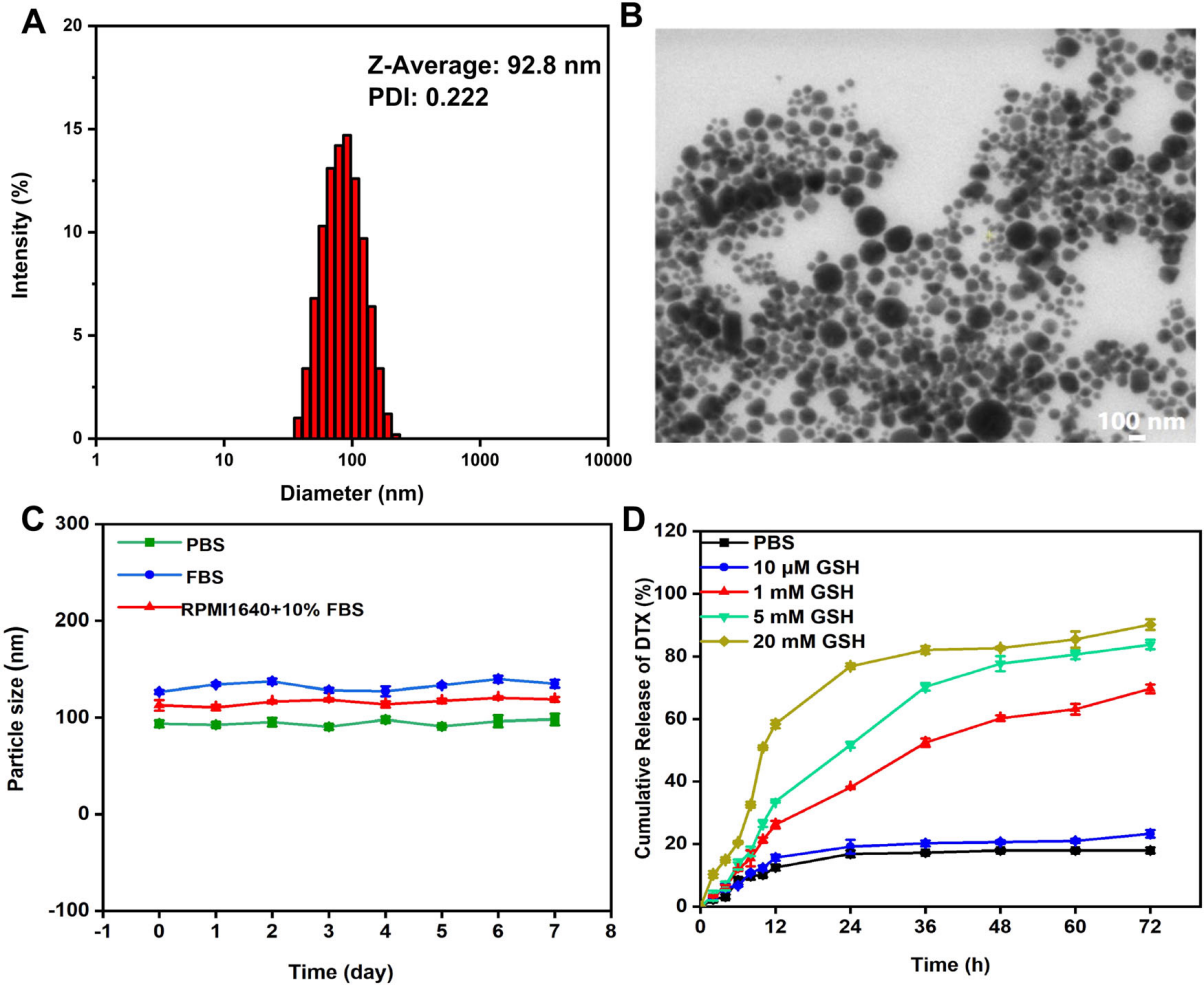
The characterization of poly-TTG-SS NPs. The average diameter of the NPs by DLS (**A**), TEM image (**B**) Scale bar: 100 nm. The stability in different media (**C**). The in vitro DTX release behavior of drug-loaded poly-TTG-SS@DTX NPs in the presence of different concentrations of GSH (**D**)

The content of the stabilizer DSPE-PEG3400 was determined during the preparation of NPs. As shown in Table 1 and Fig. 2A, the appropriate average diameter of the NPs by DLS was 92.8 ± 2.5 nm (PDI: 0.222 ± 0.023) with a negative surface charge of −24.7 ± 5.6 mV when the stabilizer content was 20% in the poly-TTG-SS NPs. Thus, 20 wt.% of DSPE-PEG3400 was used for further studies.

**Table 1:** The particle parameters of different ratio of DSPE-PEG3400 in poly-TTG-SS NPs

Ratio of DSPE-PEG3400 in poly-TTG-SS NPs	Size ± SD (nm)	Zeta-potentia ± SD (mV)	PDI ± SD
20%	92.8 ± 2.5	−24.7 ± 5.6	0.222 ± 0.023
30%	99.8 ± 0.8	−25.7 ± 3.2	0.226 ± 0.121
40%	102.4 ± 1.2	−27.5 ± 2.2	0.229 ± 0.028
50%	97.1 ± 2.4	−36.8 ± 1.2	0.233 ± 0.022

As shown in Fig. 2B, TEM showed the spherical morphology of NPs with relatively homogeneous size distribution. The stability of NPs in the serum environment is verified by DLS. Figure 2C demonstrated that no evident hydrodynamic size change was observed after storage in PBS (pH 7.4), FBS and RPMI1640 medium containing 10% FBS over a period of 7 days. The EE and DLC efficiency of drug-loaded NPs were summarized in Table 2. As shown in Table 2, NPs displayed a remarkable higher EE and DLC efficiency (77.6–95.9%, 4.2–14.5%) when compared to the traditional nanomedicines.

**Table 2:** The DLC and EE of poly-TTG-SS@DTX NPs

Ratio of DTX in poly-TTG-SS@DTX NPs	DLC (%)	EE (%)
5%	4.2	95.9
10%	8.8	89.7
15%	11.9	86.4
20%	14.5	86.9
25%	13.3	77.6

When the ratio of DTX content was 20%, poly-TTG-SS@DTX NPs displayed the relative higher DLC and EE, which were 14.5% and 86.9%, respectively. Therefore, poly-TTG-SS@DTX NPs were prepared under the condition, which laid the foundation for the subsequent experiments.

### In vitro drug release analysis of drug-loaded poly-TTG-SS@DTX NPs

The drug-loaded poly-TTG-SS@DTX NPs designed in our study have disulfide bond, which can respond to GSH. Dialysis method was applied in order to study the in vitro drug release behavior of GSH responsive drug-loaded poly-TTG-SS@DTX NPs.

The cumulative release of DTX was 76.82 wt% after 24 h and 82.61 wt% after 48 h under the condition with high level of GSH (20 mM) (Fig. 2D). The rate of release of DTX from poly-TTG-SS@DTX NPs was reduced at low concentration of GSH (5 mM). Only 20.58% of DTX was released over 48 h from NPs under the condition of lower level of GSH (10 µM, equivalent to the level of GSH in normal extracellular matrix), which was close to that in PBS (17.96 wt%).

### Cell uptake

**Fig. 3:**
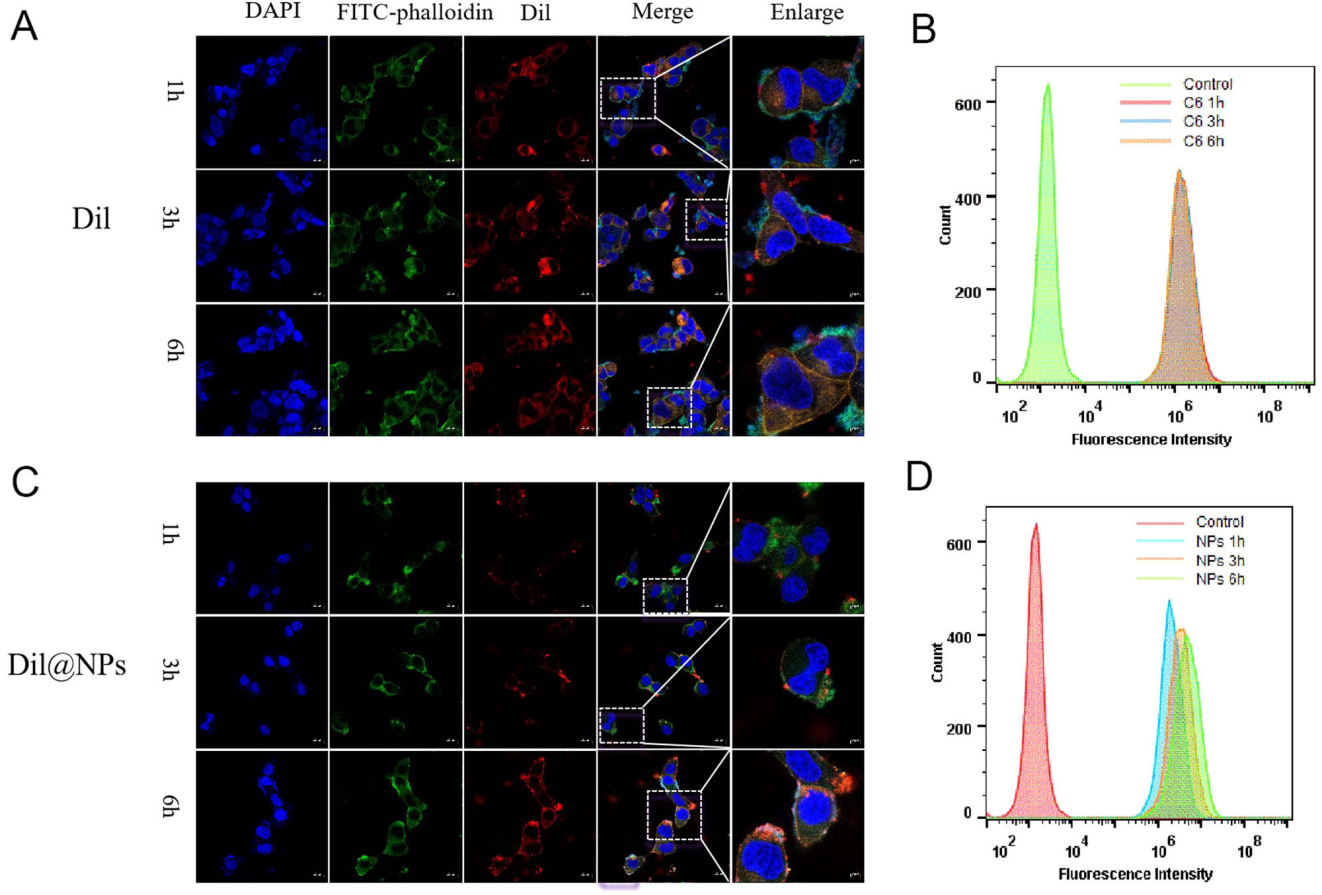
Cellular uptake of poly-TTG-SS NPs in prostate cancer cells. The direct cellular uptake of Dil (**A**) and poly-TTG-SS@Dil NPs (**C**) in C4-2 cells by CLSM. The cellular uptake of C6 (**B**) or poly-TTG-SS@C6 NPs (**D**)

The fluorescent tracker C6 was used as a model drug instead of DTX, and fluorescence intensity of C6 loaded NPs and free C6 were analyzed by flow cytometry in prostate cancer cells (C4-2 cells) in order to evaluate the cell uptake of drug-loaded NPs quantitatively. The fluorescence intensity of poly-TTG-SS@C6 NPs in C4-2 cells increased gradually with the incubation time, while there was no obvious change in the fluorescence intensity of free C6 in C4-2 cells (Fig. 3B, D).

The fluorescent dye Dil was used to visualize and localize poly-TTG-SS NPs in cancer cells. It was clearly observed with CLSM that red fluorescence could be seen within the cells after co-incubating for 1 h, indicating that poly-TTG-SS@Dil NPs NPs could be uptaken by C4-2 cells (Fig. 3C). In addition, the fluorescence signals of Dil@NPs increased gradually with the incubation time (Fig. 3C). For free Dil, obvious fluorescence was observed after 1 h of co-culture, but the fluorescence intensity did not increase significantly over time (Fig. 3A).

The cellular uptake results in the human normal prostate epithelial cells were consistent with that in prostate cancer cells (Supplementary Fig. S1).

### In vitro antitumor performance

**Fig. 4:**
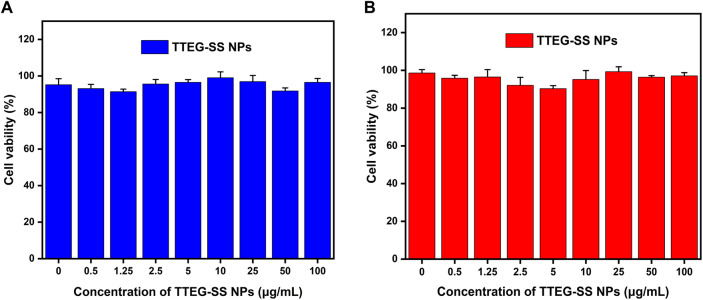
The biosafety of poly-TTG-SS NPs against 3T3 cells (**A**) and C4-2 cells (**B**)

**Fig. 5:**
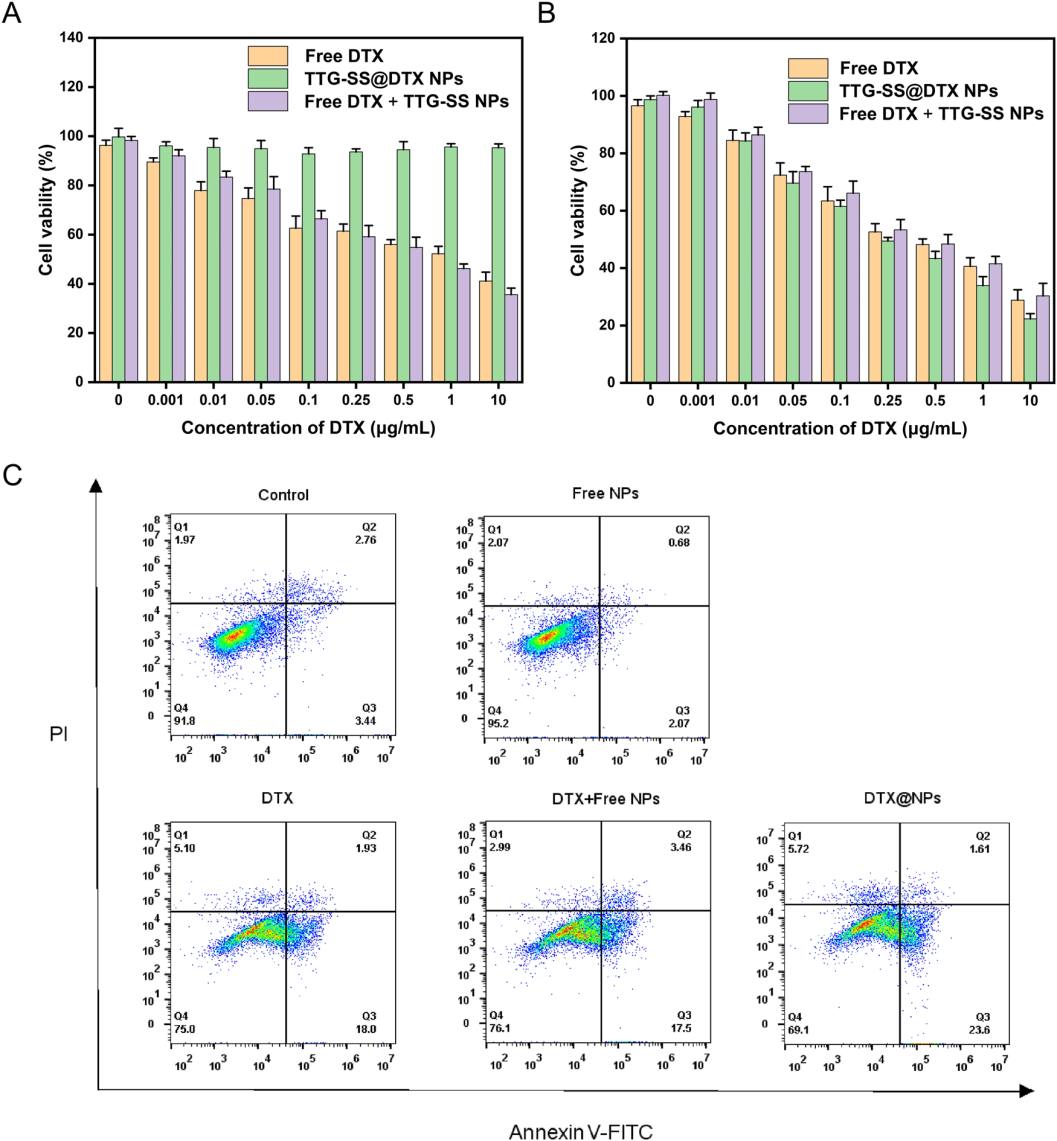
The in vitro antitumor performance of drug-loaded poly-TTG-SS@DTX NPs. The toxicity of drug-loaded poly-TTG-SS@DTX NPs, free DTX and free DTX+ poly-TTG-SS NPs against 3T3 cells (**A**) and C4-2 cells (**B**) determined by MTT. The apoptosis rate of the blank nanocarrier, free DTX, DTX loading NPs and free DTX+ poly-TTG-SS NPs detected by flow cytometry (**C**)

First, the cytotoxicity of nano-carrier poly-TTG-SS NPs against healthy cells (3T3 cells and RWPE-1 cell line) and prostate cancer cells (C4-2 cells) was analyzed by MTT assay. The cellular activity remained greater than 90% when the concentration of poly-TTG-SS NPs reached as high as 100 µg/mL treated on 3T3, C4-2 and RWPE-1 cells for 48 h (Fig. 4A, B and Supplementary Fig. S2). The above results showed that the nano-carrier poly-TTG-SS NPs have good biosafety and biocompatibility to healthy cells and prostate cancer cells. In addition, we also compared the toxicity of drug-loaded poly-TTG-SS@DTX NPs, free DTX and free DTX+ poly-TTG-SS NPs on healthy cells after 48 h (Fig. 5A and Supplementary Fig. S3). Poly-TTG-SS@DTX NPs could reduce the damage of DTX to healthy cells, which were suitable as the carrier of the chemotherapeutic drug DTX. Next, the cell toxicity of DTX loading NPs was evaluated on C4-2 cells after 48 h by comparing with DTX and DTX+ poly-TTG-SS NPs group (Fig. 5B). The cell toxicity of DTX, DTX+ poly-TTG-SS NPs and DTX loading NPs to C4-2 cells tended to enhance progressively with the elevated administration of drug concentration. The killing effect of DTX loading NPs group on C4-2 cells was close to or even stronger than that of free anti-tumor drug and free DTX combined with the blank nano-carrier (Fig. 5B).

The apoptosis rate of the blank nano-carrier, free DTX, DTX loading NPs and free DTX+ poly-TTG-SS NPs were detected by flow cytometry to further verify the anti-tumor effect of poly-TTG-SS@DTX NPs on C4-2 cells. The results displayed that the apoptosis degree of poly-TTG-SS NPs group (2.75%) was even smaller that of the blank control group (about 6.20%), while the poly-TTG-SS@DTX NPs group resulted in a stronger apoptosis effect (25.21%) compared with the free DTX group (19.93%) and free DTX+ poly-TTG-SS NPs group (20.96%) (Fig. 5C).

## Discussion

In our study, GSH-responsive poly-TTG-SS was designed and synthesized due to high levels of GSH in prostate cancer cells. The synthesis process of poly-TTG-SS was the simple polymerization reaction. The disappearance of skeletal vibration of sulfydryl demonstrated that the disulfide bond was successfully synthesized. The poly-TTG-SS NPs and drug-loaded NPs were prepared by nanoprecipitation method, which is a classic and simple method for the preparation of polymer NPs encapsulating hydrophobic drug [18]. DSPE-PEG is amphiphilic with good biocompatibility and biodegradability, which could improve the stability and drug encapsulation efficiency of nano-carriers [19]. DSPE-PEG3400 was used as the stabilizer during the preparation of NPs in our study. The particle size and surface charge of NPs are two critical factors for biological effects, including cellular uptake, toxicity, and solubility [20]. Our results revealed that poly-TTG-SS could self-assemble to form NPs characterized by nanosized diameter (92.8 ± 2.5 nm) and negatively charged surface charge of NPs (−24.7 ± 5.56 mV) with DSPE-PEG3400 (20 wt.%). Thus, 20 wt.% of DSPE-PEG3400 was used for further studies. The appropriate nanosized diameter made them suitable for tumor accumulation through EPR effect. The TEM pictures showed the spherical morphology of NPs with relatively homogeneous size distribution. The results of stability for poly-TTG-SS NPs proved that the nanoprobes have the excellent physiological stability, which could prolong blood circulation time. Drug-loaded NPs displayed a relatively higher EE and DLC efficiency (77.6–95.9%, 4.2–14.5%) when compared to the traditional nanomedicines [21, 22]. Traditional encapsulation approaches suffer from poor EE (~15%) [21] and DLC (generally less than 10%) [22]. In our study, 20% of the DTX content was chosen with the best DLC and EE (14.5% and 86.9%, respectively).

**Scheme 1:**
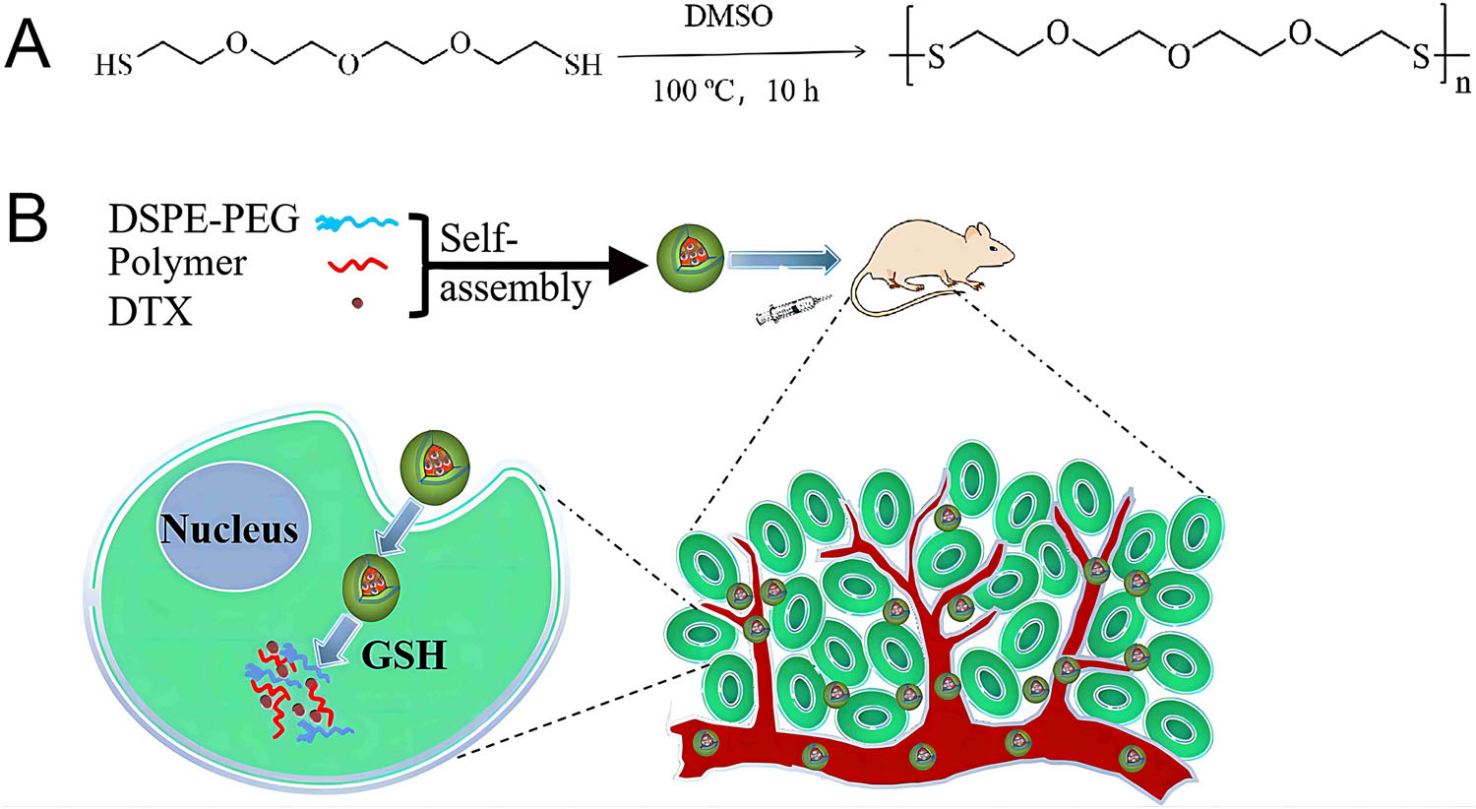
Schematic illustration of the preparation of GSH-sensitive poly-TTG-SS (**A**) and poly-TTG-SS@DTX NPs (**B**)

In vitro drug release behavior of GSH responsive drug-loaded poly-TTG-SS@DTX NPs indicated that drug release increased in the redox tumor microenvironment, which could avoid damage of DTX to normal cells in the normal physiological environment (Scheme 1).

The cell uptake results indicated that poly-TTG-SS@Dil NPs tended to be taken up with time progressively. For free Dil, the fluorescence intensity did not increase significantly over time. The reason might be the different entry mode. The entrance of NPs into cells were mainly through endocytosis, while the entrance of free Dil into cells through diffusion. Both of FCM and CLSM results indicated that the drug loading NPs were continuously and effectively taken up by prostate cancer cells (C4-2 cells) and accumulated in the cells, which provided a good opportunity for the successful delivery of DTX into prostate cancer cells by drug-loaded poly-TTG-SS@DTX NPs. The same cell uptake results of poly-TTG-SS NPs by prostate cancer cells have been found in the human normal prostate epithelial cells.

The biosafety and biocompatibility of nano-carriers are commonly used as one of the evaluation indicators for nanomedicine delivery systems. The MTT results indicated that the blank nano-carrier poly-TTG-SS NPs showed good biological compatibility to healthy cells and prostate cancer cells. The killing effect of DTX loading NPs group on C4-2 cells was close to or even stronger than that of free anti-tumor drug and free DTX combined with the blank nano-carrier. This may be related to the sustained and rapid release of DTX by the drug-loaded poly-TTG-SS@DTX NPs in response to GSH after entering C4-2 cells. The apoptosis results also showed that poly-TTG-SS NPs have good biocompatibility to cells, and poly-TTG-SS@DTX NPs could inhibit growth and induce apoptosis of prostate cancer cells effectively.

## Conclusion

In our study, GSH-responsive poly-TTG-SS was successfully synthesized by the simple polymerization reaction. Poly-TTG-SS@DTX NPs were self-assembled in water. Drug release experiments in vitro showed that drug-loaded poly-TTG-SS@DTX NPs could respond well to GSH, resulting in rapid and sustained drug release of DTX under physiological conditions. MTT cytotoxicity test and flow cytometry showed that poly-TTG-SS@DTX NPs had good compatibility to healthy cells and strong anti-tumor effect in vitro. In conclusion, poly-TTG-SS@DTX NPs may provide a new therapeutic strategy for the chemotherapy of prostate cancer.

### Supplementary information

Supplementary Figure S1

Supplementary Figure S2

Supplementary Figure S3

Supplementary Figure legends
