# Supplementary figures and images for: Tumor-microenvironment responsive nano-carrier system for therapy of prostate cancer
Source: J Mater Sci Mater Med. 2023 Sep 21;34(10):46. doi: 10.1007/s10856-023-06749-9 (PMC10514162; doi:10.1007/s10856-023-06749-9)

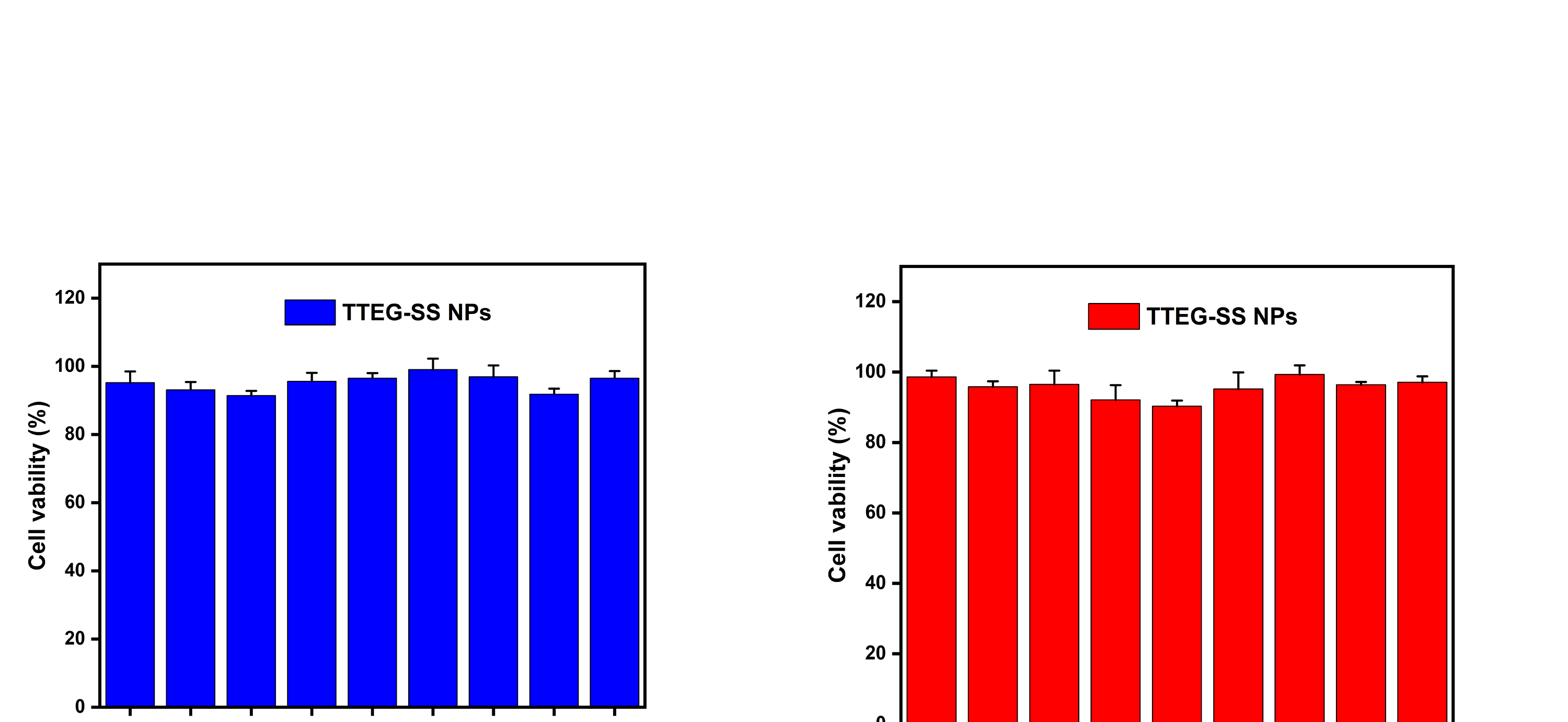

Supplement: Supplementary file 1 — Supplementary Figure S1 [file 10856_2023_6749_MOESM1_ESM.tif]
