# Supplementary material for: Tumor-microenvironment responsive nano-carrier system for therapy of prostate cancer
Source: J Mater Sci Mater Med. 2023 Sep 21;34(10):46. doi: 10.1007/s10856-023-06749-9 (PMC10514162; doi:10.1007/s10856-023-06749-9)
Supplement: Supplementary file 4 — Supplementary Figure legends [file 10856_2023_6749_MOESM4_ESM.docx]

**Supporting Information**

**Figure S1.** Cellular uptake in RWPE-1 cells treated with Dil (A) or Dil-loaded NPs (B) for 1 h, 3 h and 6 h analyzed using CLSM; Cellular uptake in RWPE-1 cells treated with C6 (C) or C6-loaded NPs (D) for 1 h, 3 h and 6 h analyzed using FCM;

**Figure S2.** Biosafety of poly-TTG-SS in RWPE-1 cells within 48 h, as determined using the MTT assay;

**Figure S3.** The cellular toxicity of drug-loaded poly-TTG-SS@DTX NPs, free DTX and free DTX + poly-TTG-SS NPs against RWPE-1 cells.
